# Involvement of AMP-activated Protein Kinase (AMPK) in Regulation of Cell Membrane Potential in a Gastric Cancer Cell Line

**DOI:** 10.1038/s41598-018-24460-6

**Published:** 2018-04-16

**Authors:** Lin Zhu, Xiao-jian Yu, Sheng Xing, Feng Jin, Wei-Jun Yang

**Affiliations:** College of Life Sciences, Zhejiang University, Hangzhou, 310058 People’s Republic of China

## Abstract

Membrane potential (V_mem_) is a key bioelectric property of non-excitable cells that plays important roles in regulating cell proliferation. However, the regulation of V_mem_ itself remains largely unexplored. We found that, under nutrient starvation, during which cell division is inhibited, MKN45 gastric cancer cells were in a hyperpolarized state associated with a high intracellular chloride concentration. AMP-activated protein kinase (AMPK) activity increased, and expression of cystic fibrosis transmembrane conductance regulator (CFTR) decreased, in nutrient-starved cells. Furthermore, the increase in intracellular chloride concentration level and V_mem_ hyperpolarization in nutrient-starved cells was suppressed by inhibition of AMPK activity. Intracellular chloride concentrations and hyperpolarization increased after over-activation of AMPK using the specific activator AICAR or suppression of CFTR activity using specific inhibitor GlyH-101. Under these conditions, proliferation of MKN45 cells was inhibited. These results reveal that AMPK controls the dynamic change in V_mem_ by regulating CFTR and influencing the intracellular chloride concentration, which in turn influences cell-cycle progression. These findings offer new insights into the mechanisms underlying cell-cycle arrest regulated by AMPK and CFTR.

## Introduction

Gastric cancer is one of the leading causes of cancer-related mortality worldwide. In 2015, over 750,000 people died of gastric cancer^1^. Despite advances in diagnostic tools and treatments, the prognosis of gastric cancer patients remains particularly poor, with an overall 5 year survival rate of approximately 20%^2^. Therefore, understanding the regulatory mechanisms that govern cancer cell proliferation, differentiation, migration, and survival is crucial for the development of new, targeted, and more effective therapeutic approaches.

Membrane potential (V_mem_), a key bioelectric property of non-excitable cells, plays functional roles in cellular processes such as proliferation, differentiation, and migration^3^. V_mem_ refers to the voltage gradient across the plasma membrane that results from the discrepancy in ion concentrations between the cytoplasm and the extracellular environment, and it arises from active and passive ion transport through numerous channels in the cell membrane, each of which has a distinct ion selectivity and permeability^3–5^. Cells are called ‘depolarized’ when V_mem_ becomes less negative, and ‘hyperpolarized’ when the potential becomes more negative^3,6^. Sodium, potassium, calcium, and chloride are the major ionic gradients across the cell membrane. In contrast to Na^+^ and Ca^2+^, most cell membranes are more permeable to potassium and chloride ions^7^. Based on the voltage gradients and ion distributions across the cell membrane, the inflow of cations such as sodium and calcium and/or the outflow of intracellular chloride anions can induce depolarization^7^.

Chloride channels, the most abundant anion in all organisms, are believed to contribute to V_mem_, and to maintain intracellular pH and cell volume^8^. The chloride current plays important roles in multiple cellular processes, including the cell cycle and proliferation^9^. Due to the chloride concentration distribution across the plasma membrane, the opening of a passive chloride flux pathway will drive an influx of chloride down its electrochemical gradient^7^. Cystic fibrosis transmembrane conductance regulator (CFTR), an ATP-gated chloride channel, is expressed in the apical cell membrane of chloride-secreting epithelial cells^10^. CFTR is not only a secretory chloride channel, but also acts as a conductance regulator, coordinating an ensemble of ion fluxes across the cell membrane^11,12^. A wide variety of membrane transport proteins are modulated by CFTR, including the epithelial sodium channel (ENaC)^13^, the outwardly rectifying chloride channel^14^, sodium/hydrogen exchanger^15^, calcium-activated chloride channels^16^, aquaporin 9 water channel^17^, and anion exchanger^18^. Thus, CFTR is an important determinant of the fluctuation of V_mem_.

V_mem_ levels are tightly related with mitosis, DNA synthesis, and other events related to cell proliferation. Dividing cells, especially rapidly dividing cancer cells, are relatively depolarized, whereas non-dividing and quiescent cells, such as terminally differentiated somatic cells, are relatively hyperpolarized^3,19,20^. Several studies confirm that V_mem_ modulation can stimulate or inhibit proliferation in a predictable way. In 1960’s, Clarence D. Cone Jr. first reported that sarcoma cells undergo a transient hyperpolarization before entering mitosis, followed by rapid depolarization during M phase, suggesting that V_mem_ varies throughout the cell cycle^21^. Further, hyperpolarization reversibly blocks DNA synthesis and mitosis. Hyperpolarization to −75 mV induces a complete mitotic block in Chinese hamster ovary cells, but cell division can be resumed by depolarization to −10 mV^22^. Moreover, sustained depolarization can induce DNA synthesis and mitosis in mature neurons, mouse spleen lymphocytes, and muscle cells^23–25^.

Emerging data suggest that V_mem_ and ion channels have functional roles in cancer progression, thus displaying prognostic value in clinical cancer therapy^26,27^. In the Xenopus model, depolarization of embryonic cells by manipulating the activity of native glycine receptor chloride channel induces these drastic changes in melanocyte behavior via a serotonin-transporter-dependent increase of extracellular serotonin^28^. Ivermectin, an antiparasitic agent, induces cell death and delays tumor growth through a mechanism related to chloride-dependent membrane hyperpolarization in leukemia cells^29^. In addition, V_mem_ also emerged as regulators of stem cell behavior and developmental processes^4,30,31^. V_mem_ hyperpolarization is found required for differentiation of human mesenchymal stem cell (hMSC). Further, V_mem_ depolarization would reduce the differentiated phenotype of hMSC-derived cells and improves their transdifferentiation ability, but does not fully recover the genetic profile of stem cell-like^32,33^. Pharmacologic or genetic perturbation of endogenous H+/K+- ATPase randomized the sided pattern of asymmetri-cally expressed genes and induced organ heterotaxia. Thus, LR asymmetry determination depends on a very early differential ion flux created by H+/K+-ATPase activity^34^.

V_mem_ and ion channel processes are highly energy-intensive. Conversely, both energy production and energy consumption are profoundly influenced by transport processes across the cell membrane^35,36^. Adenosine mono-phosphate (AMP)-activated protein kinase (AMPK) is a ubiquitously expressed serine/threonine kinase protein complex that acts as a sensor of cellular metabolic state, and thus plays a pivotal role in many metabolic processes^37^. AMPK is activated by elevated AMP:ATP ratio and phosphorylation of the α-subunit at Ser172 by upstream kinases such as LKB1 and calmodulin-dependent protein kinase kinase-β^38,39^. AMPK is involved in multiple cellular processes, including stimulation of autophagy, inhibition of cell growth and proliferation, and stress responses^40^. In addition, AMPK regulates a wide variety of ion channels, coupling ion transport processes to cellular stress and metabolic state^35,36,41^.

AMPK directly or indirectly alters the activities and expression levels of diverse potassium, sodium, calcium, and chloride channels. As a kinase, AMPK inhibits the activity of ion channel proteins such as H^+^-ATPase, Kir6.2, and BK_Ca_ by direct phosphorylation^42–44^. On the other hand, direct phosphorylation by AMPK stimulates activation of Kv2.1^45^. In addition to its direct functions, AMPK stimulates Nedd4–2 (neuronal precursor cells expressed developmentally down-regulated), an ubiquitin ligase that marks ion channel proteins such as ENaC, Kir2.1, and Kv7.1 for clearance from the cell membrane and subsequent degradation^46–48^. Accordingly, AMPK is involved in the regulation of chloride secretion, specifically by decreasing the activity of chloride channel proteins implicated in epithelial transport and cell volume regulation^35^. For instance, AMPK inhibits the chloride channel CFTR by direct phosphorylation, thereby blocking CFTR-dependent intracellular chloride efflux^10,12^.

Increasing evidence indicates that AMPK provides an essential link between cellular energy metabolism and ion transport processes. To date, however, no report has provided direct evidence of a regulatory role of AMPK on V_mem_ or subsequent effects on cellular processes. In this study, we found that elevated AMPK activity induced hyperpolarization in MKN45 cells by inhibiting outflow of intracellular chloride and decreased the cell-surface expression of CFTR. Moreover, the hyperpolarization induced by AMPK suppressed the division of MKN45 cells.

## Results

### Nutrient starvation induces reversible hyperpolarization and suppression of cell division in MKN45 cells

**Figure 1:**
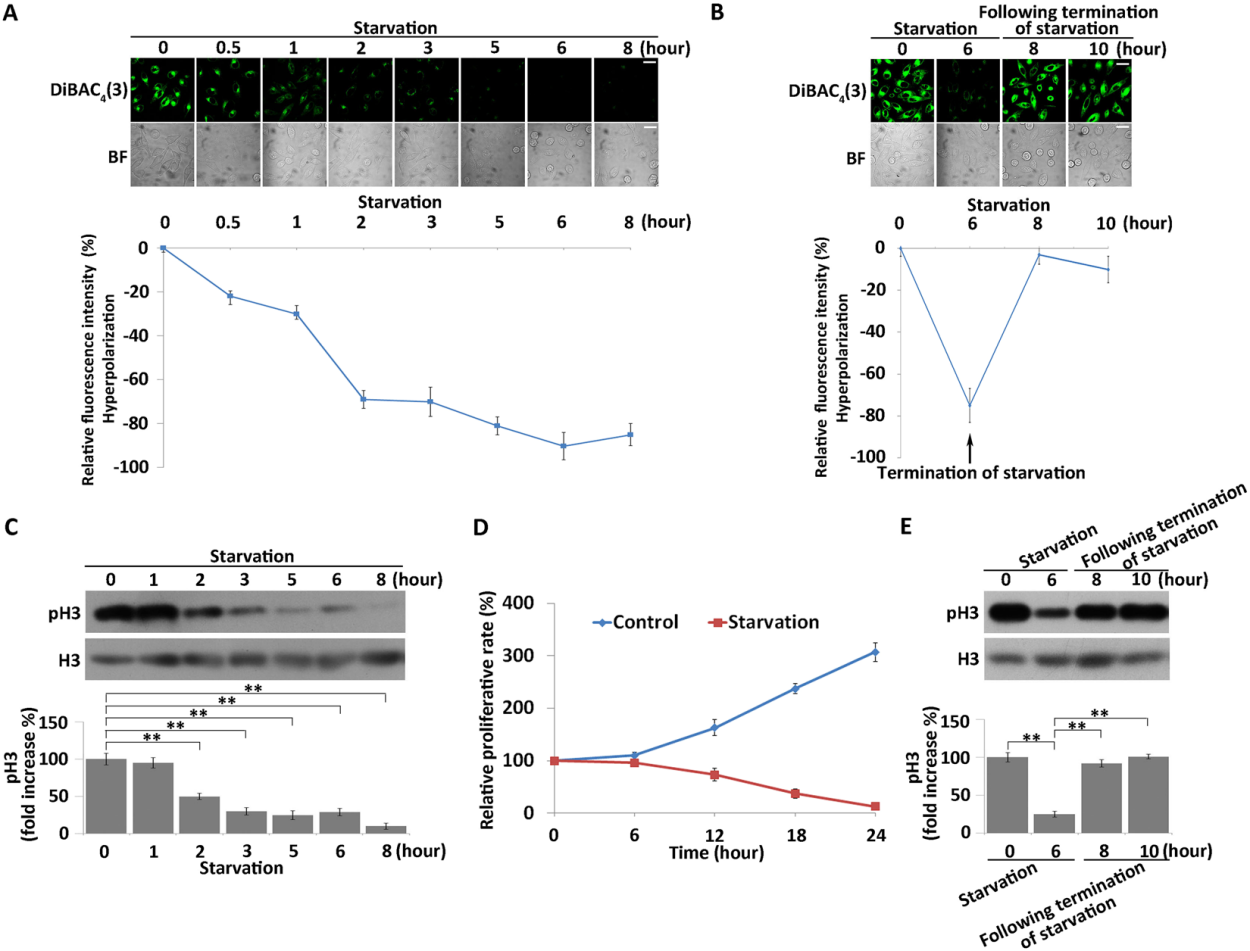
Starvation treatment induces reversible hyperpolarization and suppression of cell division. (**A**) Membrane potential of MKN45 cells after starvation treatment. Membrane potential was determined using a fluorescent bioelectricity reporter, DiBAC_4_(3) (green), as described in “*Methods*”. BF = bright field. Scale bars, 50 µm. Relative DiBAC_4_(3) fluorescence intensity changes were quantified (bottom). All data are expressed as means ± SD. (**B**) MKN45 cells were pretreated with EBSS for 6 hours, and the membrane potential of the cells following termination of starvation treatment was detected using DiBAC_4_(3) (green). BF = bright field. Scale bars, 50 µm. Relative DiBAC_4_(3) fluorescence intensity changes were quantified (bottom). All data are expressed as means ± SD. (**C**) Western blot analysis of phosphorylation of H3 after starvation treatment. Quantitative data of optical band densitometry are shown. All data are expressed as means ± SD. **P < 0.05. (**D**) Proliferation rates of MKN45 cells after starvation treatment were assessed by CCK-8 assay. All data are expressed as means ± SD. (**E**) Western blot analysis of expression and phosphorylation of H3 after termination of starvation. Means ± SD. **P < 0.05.

To assess the effect of nutrient starvation on V_mem_, we cultured MKN45 cells in Earle’s balanced salt solution (EBSS, lacking glucose and free amino acids) without FBS to induce nutrient starvation. V_mem_ was measured using DiBAC_4_(3), an anionic and membrane potential–sensitive dye. As shown in Fig. 1A, starvation treatment of MKN45 cells led to an immediate and significant decrease in the fluorescence intensity of DiBAC_4_(3) in a time-dependent manner, indicating that cells were hyperpolarized during nutrient starvation. Furthermore, nutrient-starved cells rapidly depolarized to basal level within 2 hours after the EBSS was replaced with growth medium (Fig. 1B). These results indicated that cells up-regulate V_mem_ in response to nutrient starvation.

A large body of data indicates that nutrient starvation induces cell-cycle arrest^49,50^. We evaluated the effect of nutrient starvation on cell division by Western blot to detect phosphorylation of histone H3 at serine 10 (H3S10). H3S10 levels decreased, accompanied by time-dependent hyperpolarization, with the extension of processing time, suggesting that cell division was suppressed after starvation treatment (Fig. 1C). CCK-8 assay of nutrient-starved cells revealed that cell proliferation was suppressed as expected (Fig. 1D). Flow cytometry analysis revealed that the cell cycle was arrested at S phase after starvation treatment (Fig. S1). After replacement of EBSS, phosphorylation of H3 was increased to the basal level within 2 hours, indicating that cell division resumed after starvation terminated (Fig. 1E). A flow cytometric analysis confirmed that the cell cycle progressed from S phase to G2/M phase after termination of starvation treatment (Fig. S2). These results revealed that non-dividing cells were hyperpolarized, and that cells in division were relatively depolarized, consistent with the results of previous studies.

### Nutrient starvation induces an increase in intracellular chloride concentration by suppressing CFTR in MKN45 cells

**Figure 2:**
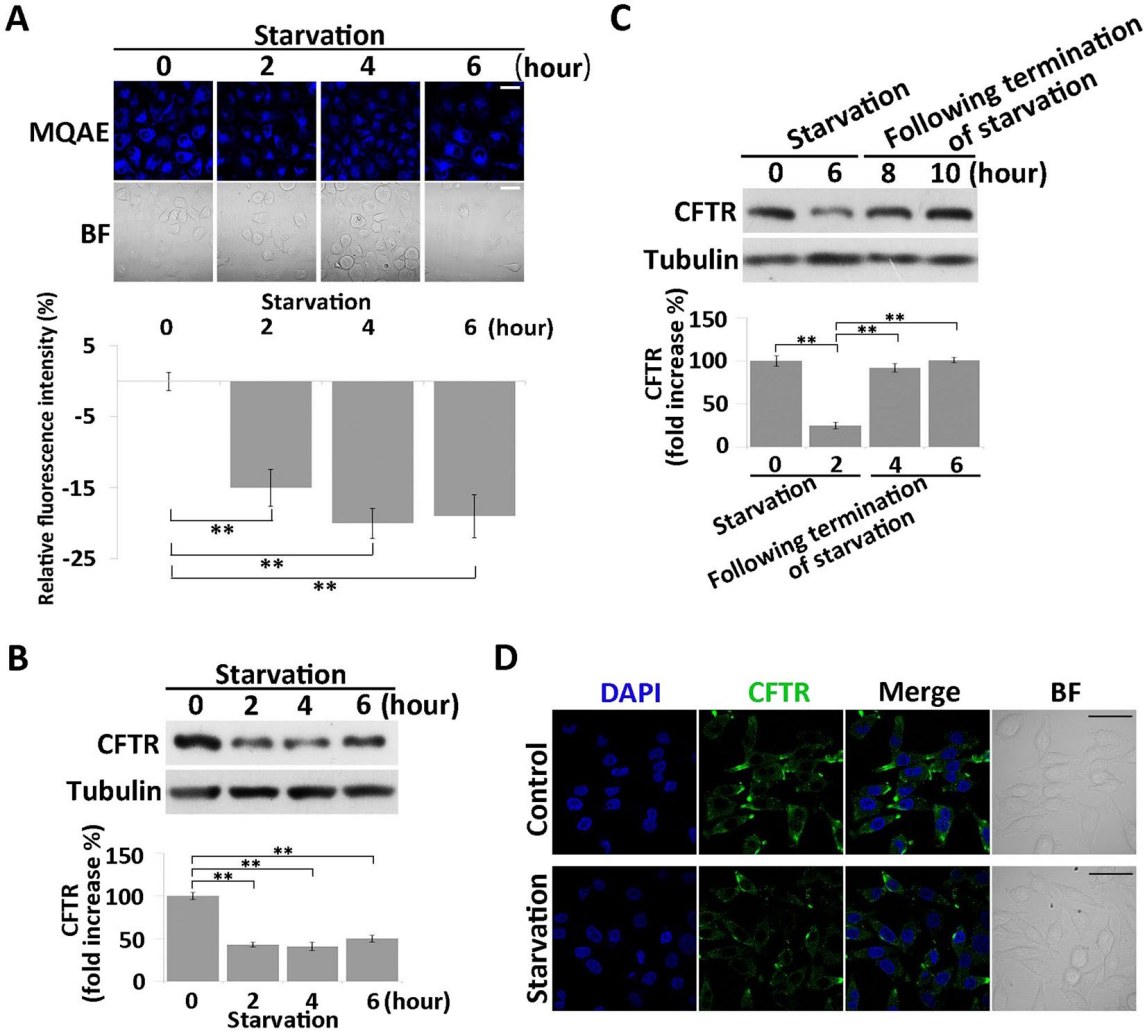
Starvation treatment induces increase of intracellular chloride concentration and suppression of CFTR. (**A**) Measurement of intracellular chloride concentration in nutrient-starved cells using the chloride-sensitive dye MQAE (blue). BF = bright field. Scale bars, 50 µm. Relative MQAE fluorescence intensity changes were quantified (bottom). All data are expressed as means ± SD. **P < 0.05. (**B**) Western blot analysis of CFTR expression in nutrient-starved cells. α-Tubulin served as a loading control. Quantitative data of optical band densitometry are shown. All data are expressed as means ± SD. **P < 0.05. (**C**) Western blot analysis of CFTR expression after termination of starvation. Means ± SD. **P < 0.05. (**D**) Immunofluorescence analysis of CFTR expression in cells nutrient-starved for 4 hours. DAPI stained the cell nuclei (blue). Green signals reflect the expression of CFTR. Scale bars, 50 µm.

To determine whether the hyperpolarization of MKN45 cells involved chloride ion, we measured intracellular chloride concentration under nutrient starvation using a chloride-selective fluorescence dye, MQAE. We found that fluorescence intensity decreased after starvation, indicating that this treatment increased the intracellular chloride concentration in these cells (Fig. 2A).

Next, we focused on the chloride ion channel CFTR, which directly controls intracellular chloride concentration. Expression of CFTR was down-regulated after starvation treatment (Fig. 2B), but returned to the basal level after starvation was terminated (Fig. 2C), indicating that expression of CFTR was suppressed during starvation. Immunofluorescence analysis revealed that the distribution of CFTR on the cell membranes decreased after starvation treatment, but this was not accompanied by an obvious decrease in the cytoplasmic level of CFTR (Fig. 2D), that which might or might not relate to the reported fragmentation of CFTR in cells into differently sized N and C terminal fragments^51^. These results suggested that nutrient-starved cells were hyperpolarized by an increase in the intracellular chloride concentration due to suppression of CFTR expression.

### Inhibition of CFTR suppresses cell division by inducing hyperpolarization in MKN45 cells

**Figure 3:**
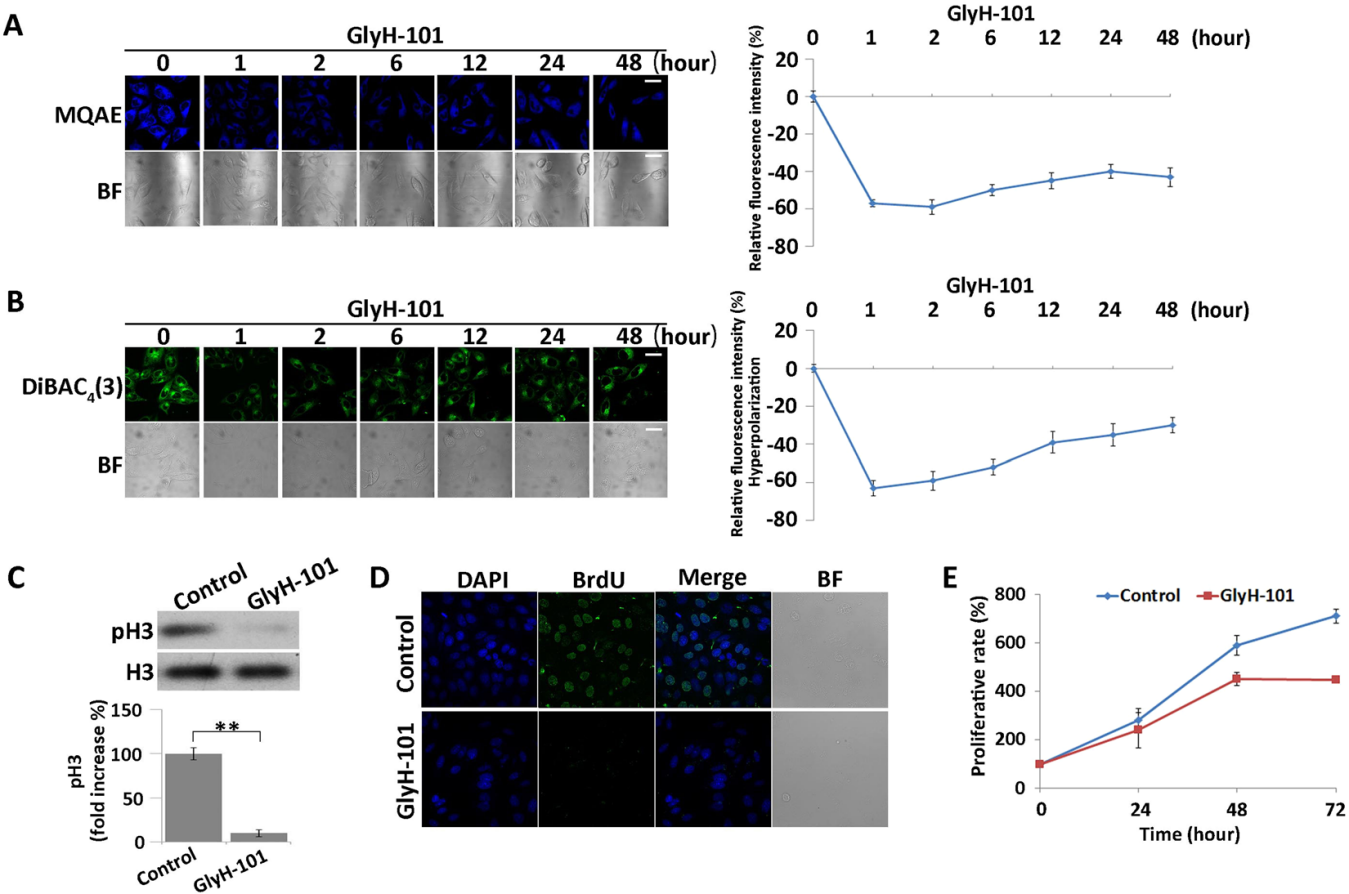
Inhibition of CFTR activity using GlyH-101 causes hyperpolarization and suppresses cell division. (**A**) Measurement of intracellular chloride concentration in GlyH-101 (10 nM)-treated cells using the chloride-sensitive dye MQAE (blue). BF = bright field. Scale bars, 50 µm. Relative MQAE fluorescence intensities were quantified (right). All data are expressed as means ± SD. (**B**) Membrane potential of cells treated with or without 10 nM GlyH-101, detected using DiBAC_4_(3) (green). BF = bright field. Scale bars, 50 µm. Relative DiBAC_4_(3) fluorescence intensity changes were quantified (right). All data are expressed as mean ± SD. (**C**) Western blot analysis of expression and phosphorylation of H3 in GlyH-101 (10 nM for 48 hours)-treated cells. Quantitative data of optical band densitometry are shown. Means ± SD. **P < 0.05. (**D**) BrdU incorporation assay and corresponding DAPI staining of GlyH-101–treated and DMSO-treated (control) cells. DAPI stained cell nuclei (blue). Red signals indicate BrdU staining. Scale bars, 50 µm. (**E**) Proliferation rates of GlyH-101 (10 nM)- and DMSO (control)-treated cells were assessed by the CCK-8 assay. All data are expressed as means ± SD.

To explore the function of CFTR in modulation of V_mem_ and cell division, we used the CFTR-specific inhibitor GlyH-101 to inhibit CFTR activity. The fluorescence intensity of MQAE decreased in GlyH-101–treated cells, indicating that inhibition of CFTR induced an increase in intracellular chloride concentration (Fig. 3A). In addition, we monitored the effects of CFTR inhibition on V_mem_. GlyH-101 treatment led to an immediate and significant decrease in DiBAC_4_(3) fluorescence intensity, suggesting that cells were hyperpolarized after CFTR inhibition (Fig. 3B).

To study the influence of hyperpolarization on cell division, we measured phosphorylation of H3 and BrdU incorporation and performed a CCK-8 assay on CFTR-inhibited cells. Western blot analysis revealed that phosphorylation of H3 was down-regulated in GlyH-101–treated cells (Fig. 3C). BrdU incorporation was observed in the control group, but no BrdU signal was detectable in GlyH-101–treated cells (Fig. 3D). The CCK-8 assay confirmed that GlyH-101 treatment inhibited proliferation (Fig. 3E). Together, these results demonstrate that inhibition of CFTR activity suppresses cell division.

### Suppression of the increase in AMPK activity prevents hyperpolarization and suppression of cell division during nutrient starvation

**Figure 4:**
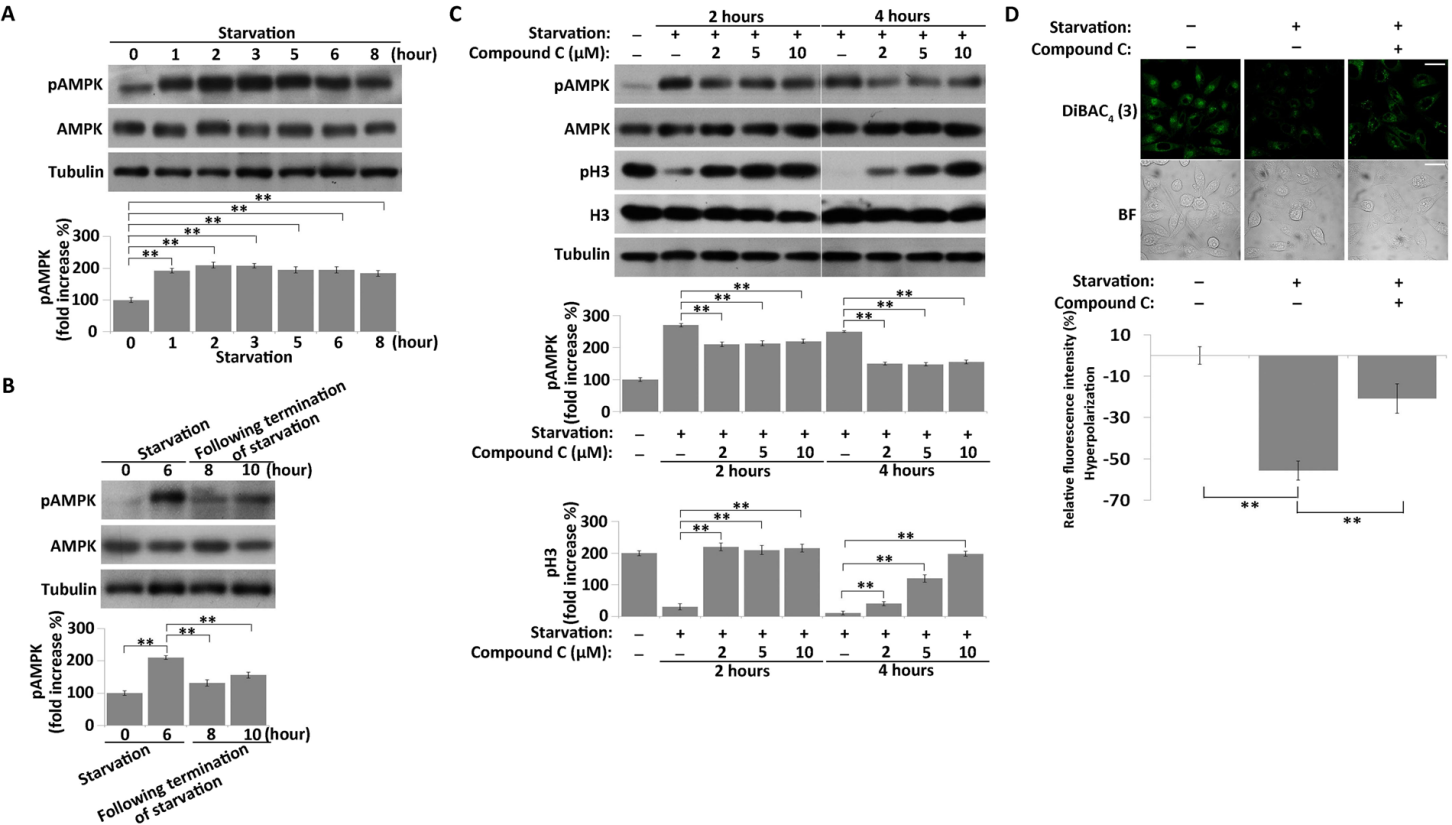
Starvation treatment induces a reversible increase in AMPK activity, and suppressing the increase in AMPK activity prevents hyperpolarization in nutrient-starved MKN45 cells. (**A**) Western blot analysis of expression and phosphorylation level of AMPK in nutrient-starved MKN45 cells. Quantitative data of optical band densitometry are shown. Means ± SD. **P < 0.05. (**B**) Western blot analysis of AMPK activity after termination of starvation. (**C**) Western blot analysis of expression and phosphorylation level of AMPK and H3 in nutrient-starved MKN45 cells, treated or not with compound C. Means ± SD. **P < 0.05. (**D**) Membrane potential of nutrient-starved cells, treated or not with 10 µM compound C for 4 hours, detected using DiBAC_4_(3) (green). BF = bright field. Scale bars, 50 µm. Relative DiBAC_4_(3) fluorescence intensity changes were quantified (bottom). All data are expressed as means ± SD. **P < 0.05.

AMPK, an energy sensor, is over-activated in response to metabolic stress^37^. To explore the regulation of V_mem_ during nutrient starvation, we monitored the phosphorylation of AMPK during and after starvation. AMPK activity significantly increased in cells hyperpolarized in response to starvation (Fig. 4A), and then decreased to the basal level after starvation was terminated (Fig. 4B). These results suggested that AMPK activity is involved in the up-regulation of V_mem_ during nutrient starvation.

To verify the involvement of AMPK, we used compound C to inhibit the phosphorylation of AMPK in response to nutrient starvation. Western blot showed that phosphorylation of AMPK increased, whereas the H3S10 decreased, in nutrient-starved cells (Fig. 4C, lane 2 and lane 6). However, in comparison with that in cells that were only starved, phosphorylation of AMPK decreased in cells subjected to combined starvation and compound C treatment (Fig. 4C, lanes 3–5 and 7–9), suggesting that the increase in AMPK activity was suppressed to a certain extent by compound C during starvation. Meanwhile, the H3S10 level was increased nearly to the normal level in cells co-treated with compound C and starvation (Fig. 4C, lanes 3–5 and 7–9), indicating that suppression of the increase of AMPK activity prevented the suppression of cell division after starvation.

Next, we monitored the effects of AMPK activity on V_mem_. The intensity of DiBAC_4_(3) signal in co-treated cells increased significantly relative to that in cells that were only starved, indicating that co-treated cells were depolarized relative to starvation-only cells (Fig. 4D). However, compound C alone did not maintain the V_mem_ of co-treated cells at the normal level (Fig. 4D), likely because compound C could not completely suppress the increase in AMPK activity during starvation (Fig. 4C). These results indicated that the hyperpolarization induced by starvation is mediated by an increase in AMPK activity, and also revealed the regulatory function of AMPK activity and V_mem_ in cell division during nutrient starvation.

### Increased AMPK activity induces hyperpolarization by suppressing CFTR, and suppresses cell division in MKN45 cells

**Figure 5:**
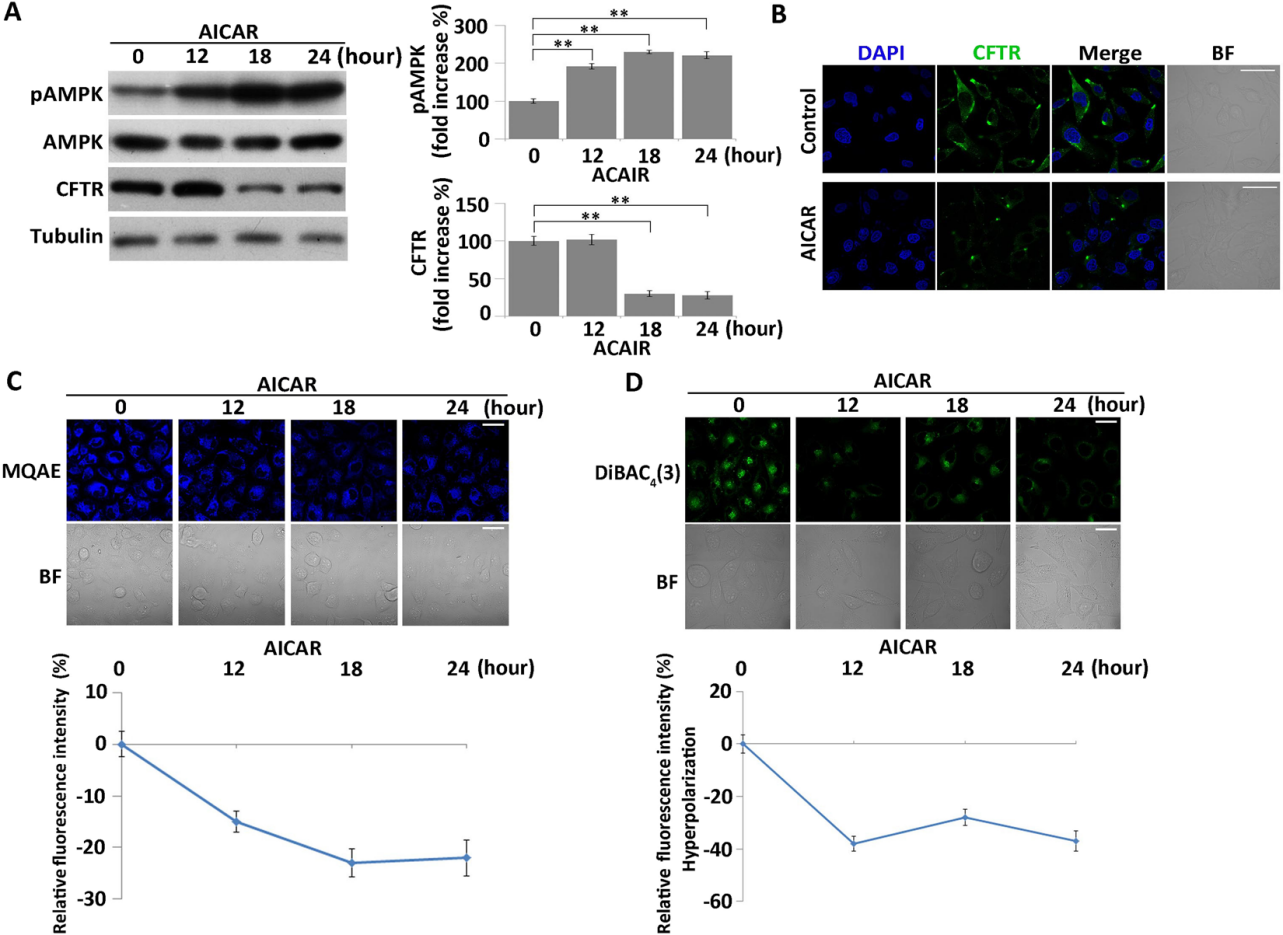
AICAR treatment suppresses CFTR and induces artificial hyperpolarization by increasing the AMPK activity. (**A**) Western blot analysis of AMPK activity and CFTR expression in AICAR-treated cells. Quantitative data of optical band densitometry are shown. Means ± SD. **P < 0.05. (**B**) Immunofluorescence analysis of CFTR expression in cells treated with AICAR for 18 hours. Cell nuclei were stained with DAPI (blue). Green signals reflect expression of CFTR. Scale bars, 50 µm. (**D**) Measurement of intracellular chloride concentration in AICAR-treated cells using the chloride-sensitive dye MQAE (blue). BF = bright field. Scale bars, 50 µm. Relative MQAE fluorescence intensities were quantified (bottom). All data are expressed as means ± SD. **P < 0.05. (**D**) Membrane potential of AICAR-treated cells was detected using DiBAC_4_(3) (green). BF = bright field. Scale bars, 50 µm. Relative DiBAC_4_(3) fluorescence intensity changes were quantified (bottom). All data are expressed as means ± SD.

To investigate the function of AMPK in regulation of V_mem_ during nutrient starvation, we used AICAR, an AMPK-specific activator, to artificially increase AMPK activity. Phosphorylation of AMPK was elevated after treatment with 1 mM AICAR, and AMPK activity increased in a time-dependent manner (Fig. 5A). To determine whether the suppression of CFTR expression was regulated by AMPK during starvation, we analyzed the expression of CFTR in AICAR-treated cells. The expression level of CFTR in MKN45 cells was down-regulated after AICAR treatment for 18 hours (Fig. 5A). Immunofluorescence analysis revealed that the distribution of CFTR on cell membranes was reduced in AICAR-treated cells (Fig. 5B). In addition, elevated AMPK activation caused an increase in intracellular chloride concentration (Fig. 5C). The fluorescent signal of DiBAC_4_(3) significantly decreased after AICAR treatment (Fig. 5D), indicating that the cells became hyperpolarized after AMPK activity increased. These findings were similar to those obtained in nutrient-starved cells, and indicated that the down-regulation of CFTR was mediated by an increase in AMPK activity during nutrient starvation.

**Figure 6:**
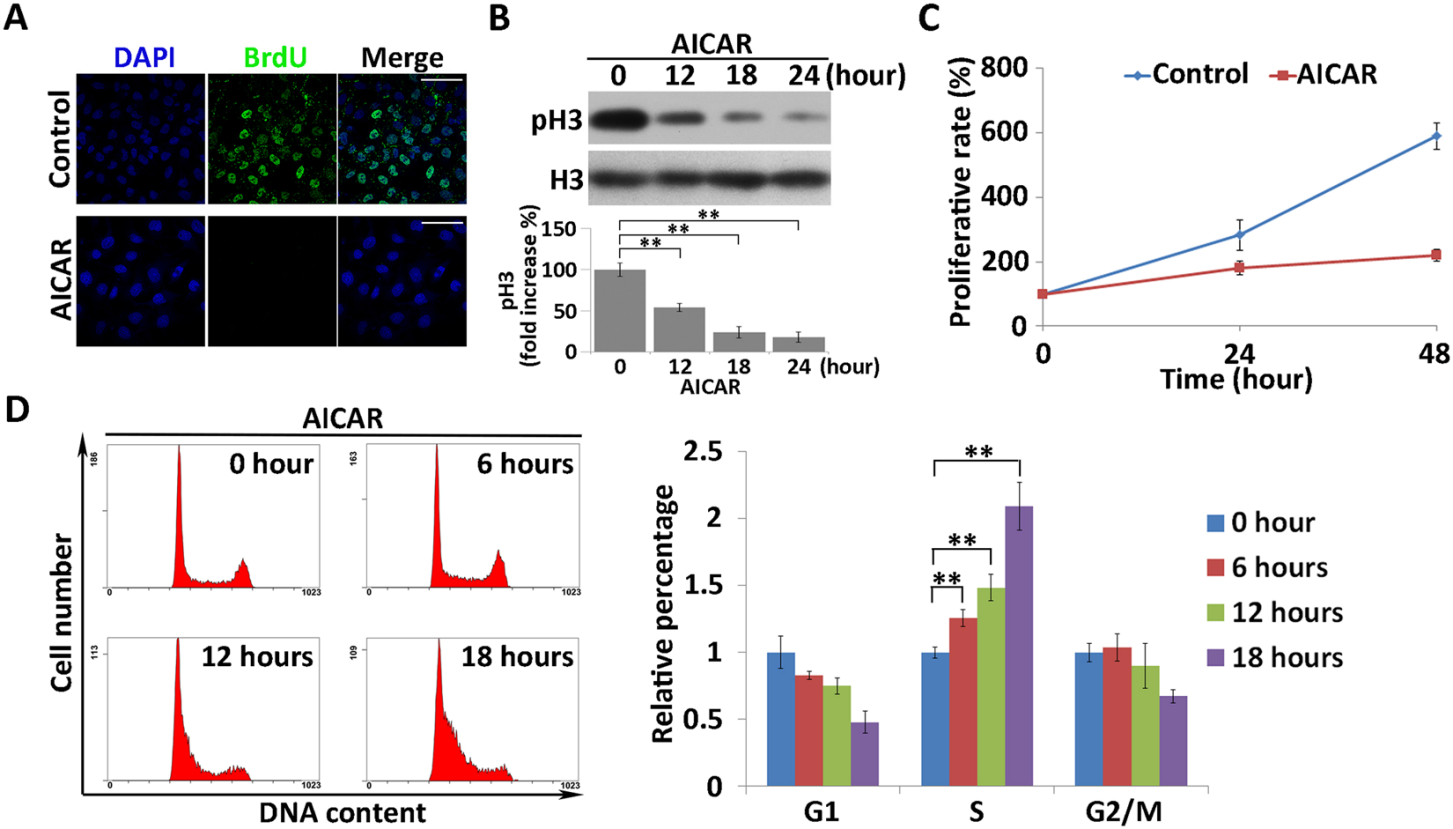
AICAR treatment inhibits cell division. (**A**) BrdU incorporation assay and corresponding DAPI staining of AICAR-treated and DMSO-treated (control) cells. Cell nuclei were stained with DAPI (blue). Red signals indicate BrdU staining. Scale bars, 50 µm. (**B**) Western blot analysis of expression and phosphorylation of H3 in AICAR-treated MKN45 cells. Quantitative data of optical band densitometry are shown. Means ± SD. **P < 0.05. **(C)** Proliferation rates of AICAR-treated cells were assessed by CCK-8 assay. All data are expressed as means ± SD. (**D**) Analysis of cell-cycle progression was performed by monitoring DNA content by flow cytometry after treatment with AICAR. Relative cell number in each phase was quantified (right). All data are expressed as means ± SD.

To study the effect of AMPK-induced hyperpolarization on cell division, we performed BrdU incorporation and CCK-8 assays on AICAR-treated cells. As shown in Fig. 6A, in contrast to the control group, no BrdU signal was observed in AICAR-treated cells, indicating that division was suppressed by the drug treatment. Phosphorylation of H3 was also diminished in AICAR-treated cells (Fig. 6B). The CCK-8 assay confirmed that cell proliferation was suppressed after AICAR treatment (Fig. 6C). Furthermore, flow cytometry analysis revealed that the cell cycle arrested at S phase after treatment with AICAR (Fig. 6D). Together, these results indicated that elevated AMPK activity suppressed the cell division.

### Hyperpolarization induced by over-activation of AMPK was reversible in MKN45 cells

**Figure 7:**
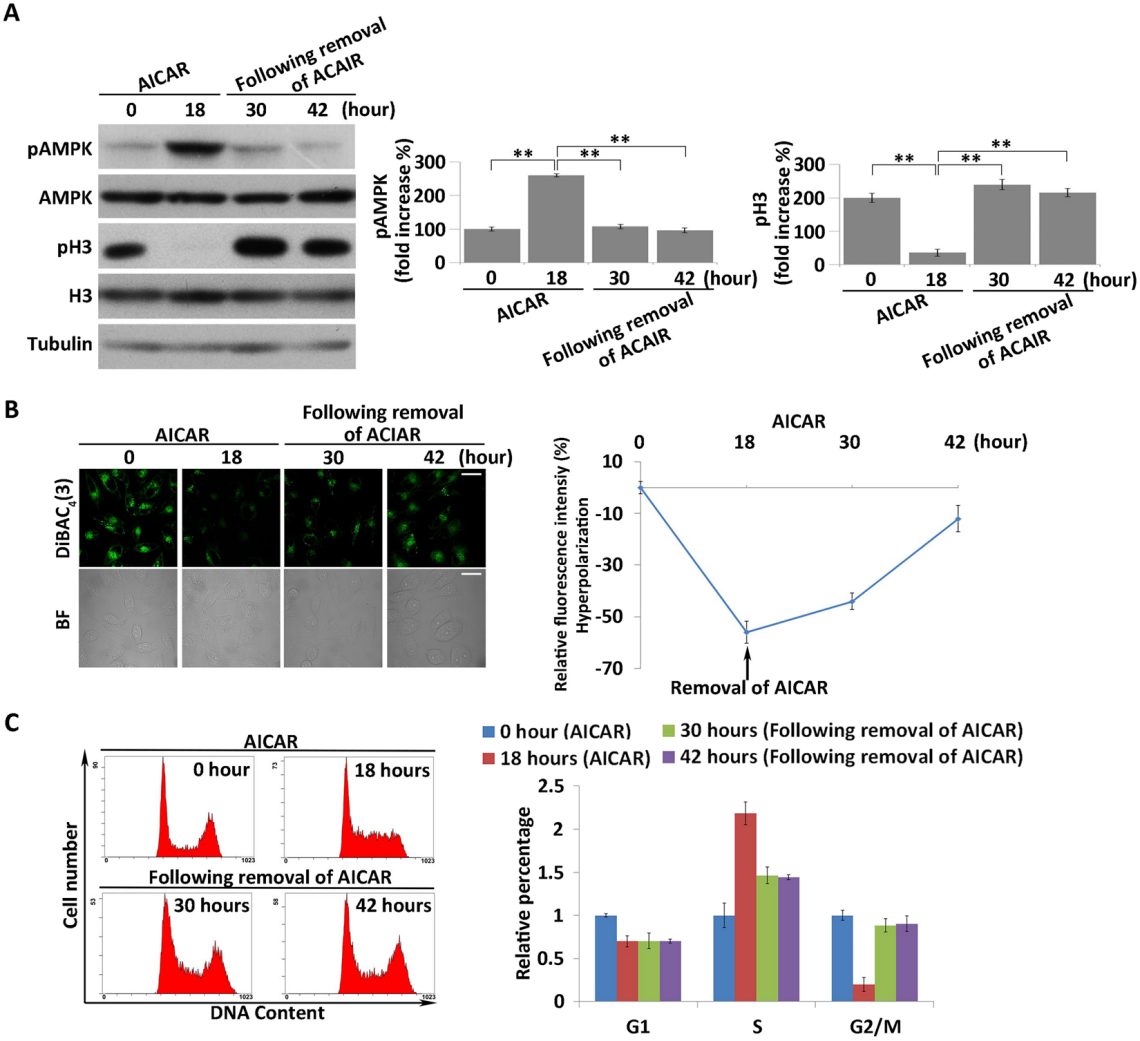
Removal of AICAR induces recovery of membrane potential and cell-cycle progression. (**A**) MKN45 cells were pretreated with 1 mM AICAR for 18 hours, and the activity of AMPK was analyzed after removal of AICAR for 12 and 24 hours. Quantitative data of optical band densitometry are shown. Means ± SD. **P < 0.05. (**B**) Membrane potential of cells after removal of AICAR for 12 and 24 hours was detected using DiBAC_4_(3) (green). BF = bright field. Scale bars, 50 µm. Relative DiBAC_4_(3) fluorescence intensity changes were quantified (right). (**C**) Cell-cycle progression was analyzed by monitoring DNA content by flow cytometry after removal of AICAR. The relative cell number in each phase was quantified (right). All data are expressed as means ± SD.

We next investigated whether over-activation of AMPK induced by AICAR treatment was reversible. Phosphorylation of AMPK decreased to the basal level within 12 hours after removal of AICAR (Fig. 7A). Accompanied by the decrease in AMPK activity, V_mem_ of the AICAR-pretreated cells became relatively depolarized after AICAR removal for 12 hours, when compared with that of the AICAR-treated cells, and the V_mem_ eventually returned to a nearly normal level (Fig. 7B). Meanwhile, flow cytometry analysis showed that the cells were released from S phase to G2/M phase after removal of AICAR, indicating that cell division resumed after AMPK activity decreased to its basal level (Fig. 7C). These results further confirmed that hyperpolarization was induced by the increase in AMPK activity.

**Figure 8:**
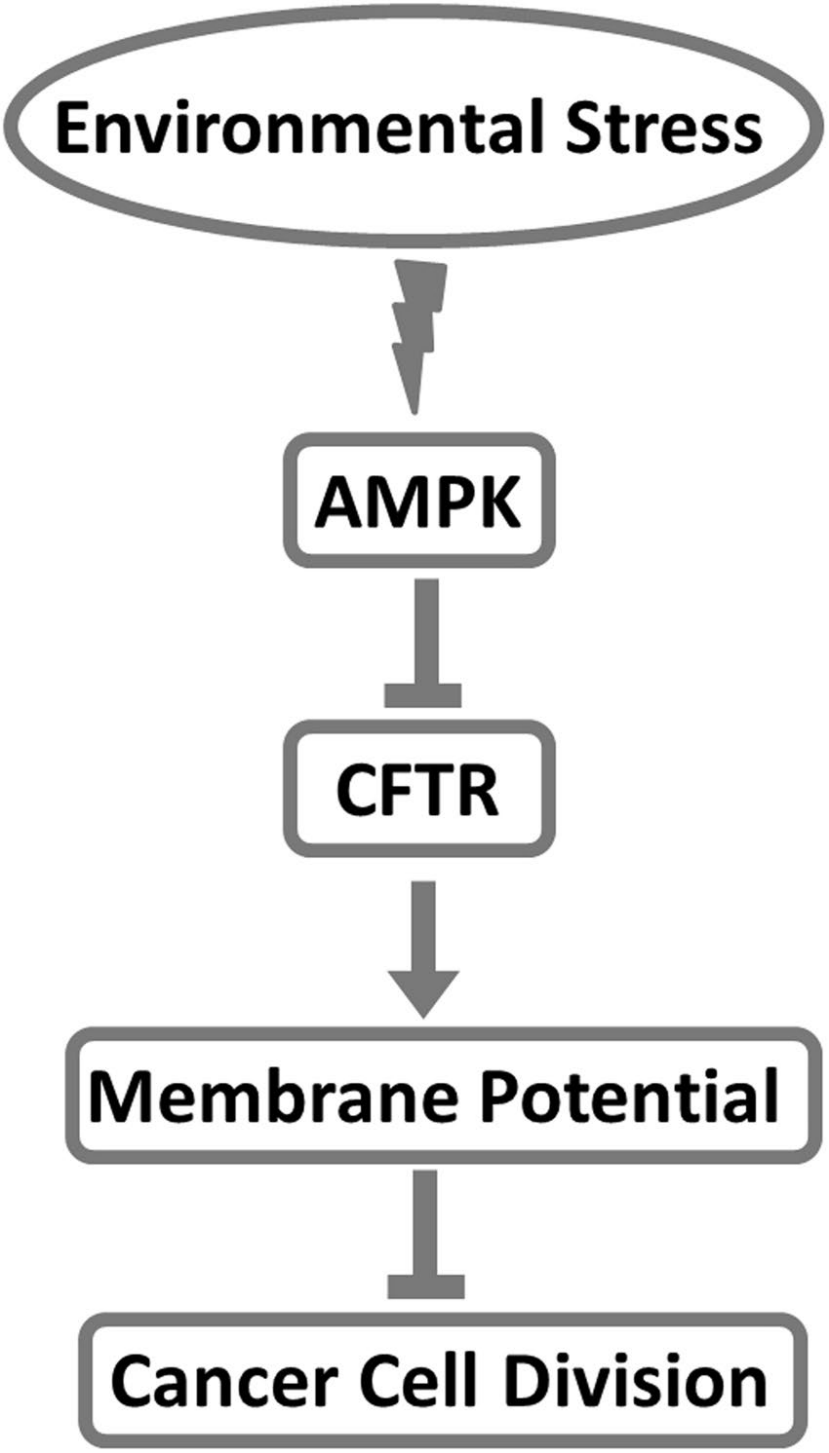
A tentative model of the involvement of AMPK in the regulation of membrane potential and cell proliferation by gastric cancer cells.

Based on these results, we propose a tentative model of nutrient starvation–mediated hyperpolarization of V_mem_ and regulation of the AMPK signaling pathway in MKN45 cells (Fig. 8). According to this model, AMPK activity increases in response to nutrient starvation, and inhibits expression and activity of CFTR. In turn, suppression of CFTR induces hyperpolarization of V_mem_, and subsequently inhibits cell division.

## Discussion

In this study, we found that nutrient starvation induced reversible hyperpolarization and cell-cycle arrest in the gastric cancer cell line MKN45. This hyperpolarization was induced by an increase in AMPK activation, which inhibited chloride efflux by suppressing CFTR expression. As a cellular energy sensor, AMPK is a therapeutic target in cancer. Acting downstream of tumor suppressor LKB1, AMPK regulates cell growth and proliferation through modulation of multiple signaling pathways, including inhibition of the mTOR pathway and stimulation of p53^52,53^. However, our findings reveal the first mechanistic details of AMPK as a powerful regulator of V_mem_, which also influences cell division. Elevation of AMPK activity induced hyperpolarization, whereas cells depolarized following reduction of AMPK activity. The changes in V_mem_ induced by AMPK activation were closely related to cell division.

Cancer cells, which exhibit sustained proliferative signaling and aberrant changes in the cell cycle, are effective models in which to study V_mem_ and the ionic regulation, or mis-regulation, of cell division. Alterations and dysfunction of V_mem_ and ion channels have been observed in a variety of cancers^9,54,55^. In general, depolarization serves as a signal that initiates mitosis and DNA synthesis. The mean V_mem_ levels of cancer cells are consistently depolarized relative to those of most normal somatic cells^3^. For example, MCF-7, a breast cancer cell line, has a V_mem_ of −9 mV at G1 phase and hyperpolarizes to about −30 mV in S phase, whereas MCF10A, a normal breast cell line, has a mean V_mem_ value between −40 and −58 mV^3,56^. Selective depolarization of cells expressing the glycine-gated chloride channel in *Xenopus* embryos results in over-proliferation of melanocytes, but this cancer-related phenotype can be reversed by expression of a hyperpolarizing channel^57^. Also in the *Xenopus* model, monitoring of a distinctly depolarized resting potential was used for early detection of tumors. Moreover, hyperpolarization itself, regardless of the types of ion channels, can prevent the formation of tumors^58,59^. Here, we found that hyperpolarized MKN45 cells were non-dividing. Further, prevention of hyperpolarization rescued the cell division, revealing that V_mem_ is a bioelectric regulator, and not merely a marker. These results confirmed the general regulation of cell division by V_mem_.

Fluctuations in chloride concentration may contribute to changes in V_mem_ during the cell cycle, and chloride currents play functional roles in the proliferation of a variety of cell types^3,9,20^. The chloride channel blocker 5-N-2-(3-phenylpropylamino) benzoic acid (NPPB) inhibits entry into S phase, and also suppresses the transition from quiescence into the cell cycle in NIH3T3 cells^60^. Moreover, several chloride channels have been implicated in cell division. For example, absence of CLC-3 chloride channel expression inhibits proliferation of vascular smooth muscle cells and glioma cells^61,62^. Fluctuations in chloride concentration also contribute to changes in cell volume for the survival of cells, and animal cells show a regulatory volume decrease by releasing intracellular Cl^-^ after osmotic swelling^63^.

Our investigation of the role of AMPK in regulation of V_mem_ revealed that chloride concentration was influenced by AMPK activity in MKN45 cells: specifically, intracellular chloride concentration significantly increased with AMPK activation, and decreased upon inactivation of AMPK, indicating that AMPK regulates chloride concentration and efflux. Specific inhibition of CFTR, a bidirectional anion channel, increased the intracellular chloride concentration, resulting in hyperpolarization. Because fluctuation in the chloride concentration is necessary for maintenance of V_mem_ and cell division, mis-regulation of chloride concentration inhibited cell division.

CFTR is an interaction partner of AMPK-α1 subunit, and its activation by the PKA pathway is inhibited by direct phosphorylation of AMPK^10,12^. In this study, we found that the level and membrane localization of CFTR significantly decreased after starvation-induced AMPK activation and AICAR treatment, suggesting the existence of a novel mechanism whereby AMPK down-regulates CFTR. AMPK is an upstream kinase of Nedd4–2, an ubiquitin ligase that targets a variety of ion channel proteins. Stimulation of Nedd4–2 by AMPK promotes endocytosis and degradation of targeted ion channel proteins such as ENaC, Kir2.1, and Orai1^46,47,64^. Otherwise, AMPK inhibits phosphatase and tensin homolog (PTEN) activity via glycogen synthase kinase 3β to suppress KATP channel trafficking^65^. A. Prince and colleagues recently proposed that PTEN binds CFTR which adds another level of complexity^66^. In addition, AMPK can inhibit activation of nuclear factor kappa B (NF-kB), thereby down-regulating NF-kB–mediated transcription of genes such as Orai1/STIM1^67–69^. Nucleoside diphosphate kinase A (NDPK-A) was found interacts with both AMPK and CFTR in airway epithelia, and NDPK-A catalytic function is required for the AMPK-dependent inhibition of CFTR activity^70^. All of these down-regulatory mechanisms provide clues regarding the reduction of CFTR expression by AMPK activation. However, not only does AMPK regulate CFTR function, but that CFTR, via metabolic-dependent interactions with AMPK, was found regulate AMPK function and subcellular localization in epithelia^71^. The results suggest that CFTR may reciprocally regulate AMPK function in our system.

In our previous study, nutrient starvation induced autophagy in MKN45 cells, and the autophagy was observed after 6 hours of starvation treatment^72^. In this study, the cells hyperpolarized rapidly within 30 minutes of starvation. The response of V_mem_ to starvation was much earlier than that of autophagy. Thus, we supposed that the cells hyperpolarized rapidly to suppress cells division and decrease metabolic level, thus resisting the starvation environment. As the starvation time extending, the autophagy level increased, which further responded to the environmental stress.

In conclusion, we found that elevation of AMPK activity down-regulates expression of CFTR in MKN45 cells, resulting in accumulation of intracellular chloride and subsequent hyperpolarization, ultimately blocking cell division. Both the AMPK signaling system and the V_mem_ regulation network are fairly complex. Our findings provide important insight into the coupling between AMPK, V_mem_ regulation, and ion channel activity. Hence, modulation of AMPK activity represents a potential therapeutic strategy for using in disorders caused by AMPK-sensitive dysregulation of V_mem_.

## Methods

### Cell Culture

The MKN45 human gastric cancer cell line was purchased from Bioleaf Biotech. MKN45 Cells were cultured in RPMI-1640 (Corning) supplemented with 10% fetal bovine serum (GIBCO), 100 IU/ml penicillin and 100 μg/ml streptomycin. All cells were cultured in a 37 °C humidified atmosphere containing 5% CO_2_ and 95% air.

### Membrane Potential Measurements

Cell membrane depolarization was measured using the membrane potential–sensitive dye bis-(1,3-dibutylbarbituric acid) trimethin eoxonol [DiBAC_4_(3), Invitrogen]. Upon transmembrane depolarization, the DiBAC_4_(3) enters the cell and binds to protein molecules, acquiring enhanced fluorescence. By contrast, fluorescence intensity is reduced when the membrane is hyperpolarized. As previously described^7,73^, after incubation with 0.5 µM DiBAC_4_(3) for 30 minutes at 37 °C, cells were subjected to time scanning using a fluorescence spectrophotometer (Thermo Scientific) with excitation at 488 nm and emission measured at 518 nm. Membrane depolarization was monitored by observing changes in the intensity of fluorescence emission of DiBAC_4_(3). All values of fluorescence image analyses were corrected for cell number and background fluorescence.

### Measurements of Chloride Concentration

Cells grown on glass coverslips were incubated with 8 mM MQAE (N-[ethoxycarbonylmethyl]−6-methoxy-quinolinium bromide, Invitrogen) for 2 hours in a cell culture incubator. After rinsing with Ringer’s Standard solution the coverslips were placed in the bottom of a perfusion chamber on the stage of an inverted microscope (Nikon). Temperature was maintained at 37 °C by heating the chamber holder and the objective separately. MQAE fluorescence was captured by using an excitation wavelength of 353 nm. The emission was measured at 460 nm using a CCD camera. All values of fluorescence image analyses were corrected for cell number and background fluorescence.

### Inhibitor Treatment

The MKN45 cancer cells were planted in 6-well plates containing medium after Trypsinized. We treated the cells with the indicated concentrations of AICAR (Selleck), compound C (Selleck), or GlyH-101 (MedChem Express) after 12 hours. The corresponding medium was replaced every day. And treated cells were harvested at the time points indicated below for Western blot analysis.

### Immunofluorescence Assay

Cells were fixed in 4% PFA at least 2 hours at room temperature or 4 °C overnight. Then washed the fixed cells in PBS. Cells were blocked in blocking buffer (1% BSA in PBS with 0.25% Triton X-100) for 30–60 minutes at room temperature. Subsequently, cells were incubated overnight at 4 °C with primary antibodies at a dilution of 1:100. The slides were washed three times with PBS and incubated with FITC-conjugated secondary antibody (Invitrogen) at room temperature. Cell nuclei were incubated with DAPI for 15 minutes.

### Western Blot Analysis

We performed the Western blot analysis as described previously^74^. Whole-cell lysates were separated by denaturing 10% SDS-PAGE gels and transferred to polyvinylidene fluoride membranes (Bio-Rad). The membranes were incubated at 4 °C overnight with specific primary antibodies after blocking in 1% blocking buffer for 1 hour, primary antibodies used were: anti-phospho-AMPK anti-phospho-H3, anti-H3 (Cell Signaling Technology), and anti-CFTR (Abcam). An anti-tubulin antibody (Sigma-Aldrich) was used as a loading control. Then the membranes were incubated in the appropriate secondary antibodies for 2 hours at room temperature. Use the BM Chemiluminescence Western Blotting kit (Roche) to detect the immunoreactive bands.

### Cell Proliferation Assay

Cell viability and proliferation were assessed using CCK-8 kit (Beyotime Biotechnology). Cells were treated with EBSS, after that, Cells were planted in 96-well plates (2000 cells/well) in triplicate for 12 hours. Use the Multiskan EX plate reader (Thermo Fisher Scientific) to quantify the viable cells by measuring absorbance at 450 nM.

### Cell-Cycle Analysis

Fixed cells were treated with RNase A (100 μg/ml; Sigma) and stained for 30 minutes with propidium iodide (50 μg/ml; Sigma) at 4 °C. Cell-cycle analysis was performed on a Beckman Coulter Flow Cytometer (FC500MCL).

### Statistical analyses

All quantitative data are expressed as means ± SD at least of three independent experiments. Comparisons between test and control values were analyzed via t test and one way ANOVA, and P values less than 0.05 were considered to be significant.

## Electronic supplementary material

Supplementary information
