# Supplementary material for: Involvement of AMP-activated Protein Kinase (AMPK) in Regulation of Cell Membrane Potential in a Gastric Cancer Cell Line
Source: Sci Rep. 2018 Apr 16;8:6028. doi: 10.1038/s41598-018-24460-6 (PMC5902619; doi:10.1038/s41598-018-24460-6)
Supplement: Supplementary file 1 — Supplementary information [file 41598_2018_24460_MOESM1_ESM.pdf]

***Supplementary Information:***

# Involvement of AMP-activated Protein Kinase (AMPK) in Regulation of Cell Membrane Potential in a Gastric Cancer Cell Line

**Lin Zhu, Xiao-jian Yu, Sheng Xing, Feng Jin and Wei-Jun Yang[1]**

College of Life Sciences, Zhejiang University, Hangzhou 310058, People's Republic of China

[1]To whom correspondence should be addressed: Wei-Jun Yang, Ph.D.

College of Life Sciences, Zhejiang University, Hangzhou, Zhejiang 310058, China

Tel./Fax: +86-571-88273176

E-mail: w_jyang@zju.edu.cn

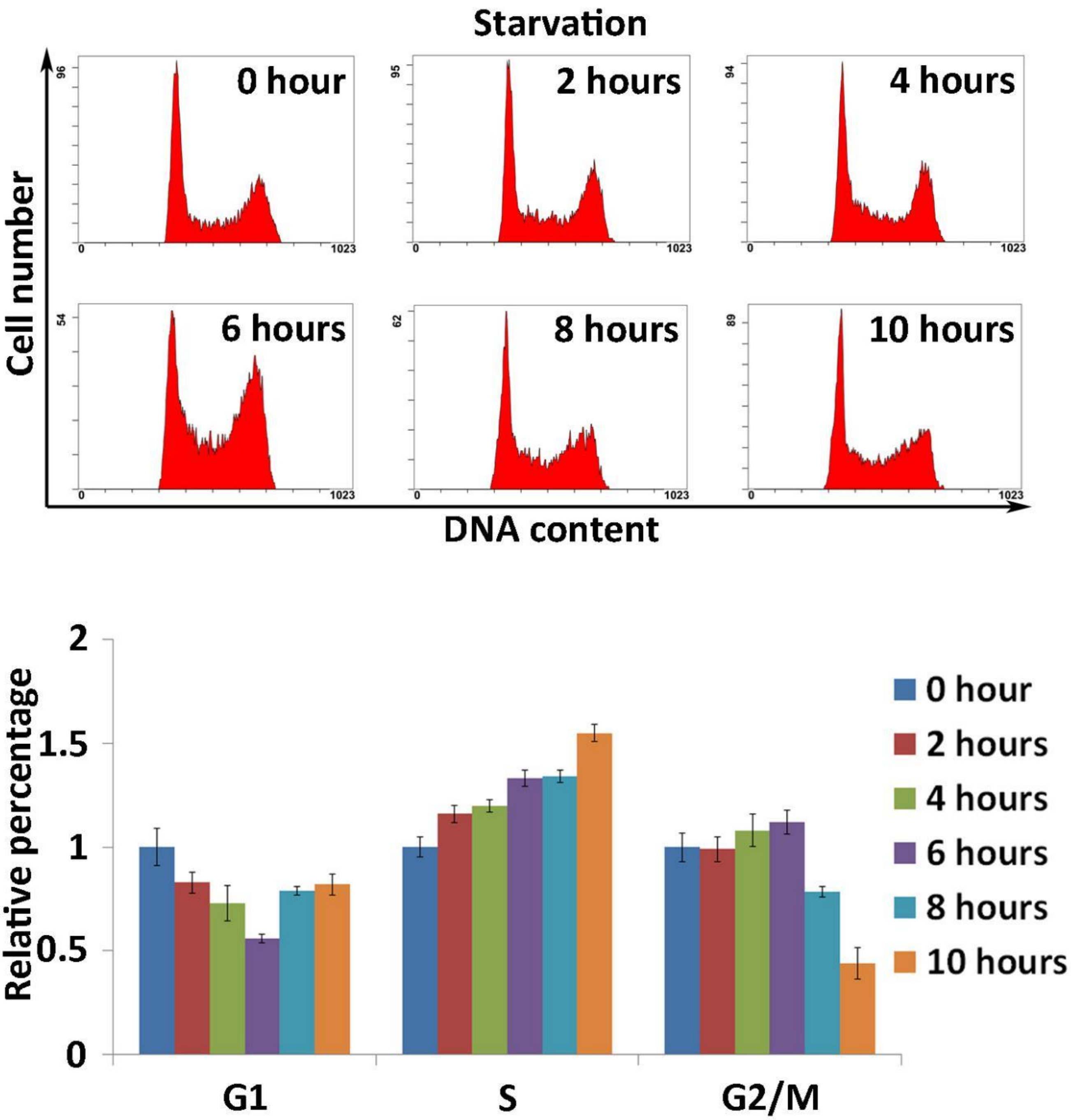


**Figure S1.** Cell-cycle progression was analyzed by monitoring DNA content by flow cytometry after starvation treatment. The relative cell number in each phase was quantified (bottom). All data are expressed as means ± SD.

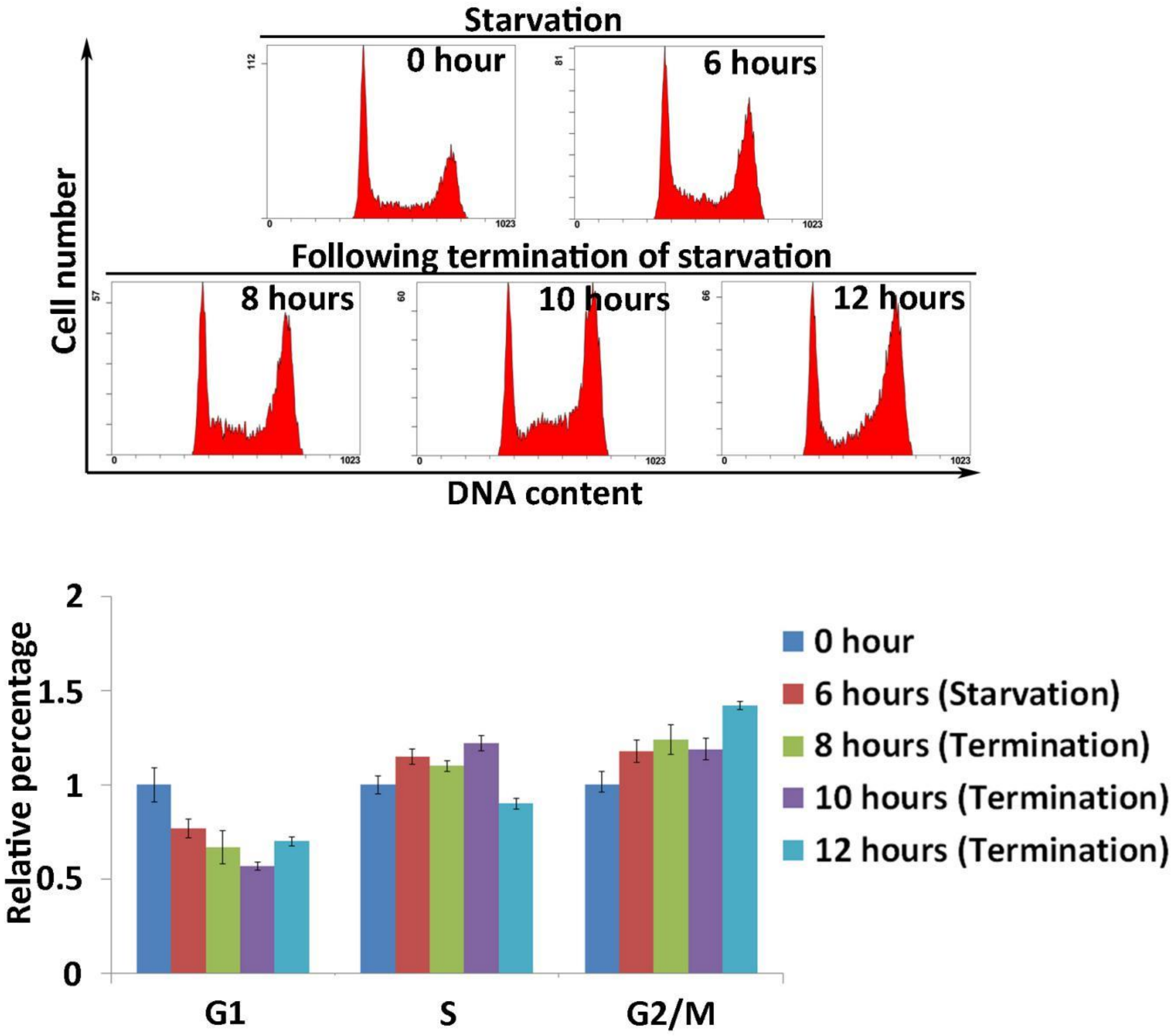


**Figure S2.** Cell-cycle progression was analyzed by monitoring DNA content by flow cytometry after termination of starvation treatment. The relative cell number in each phase was quantified (bottom). All data are expressed as means ± SD.

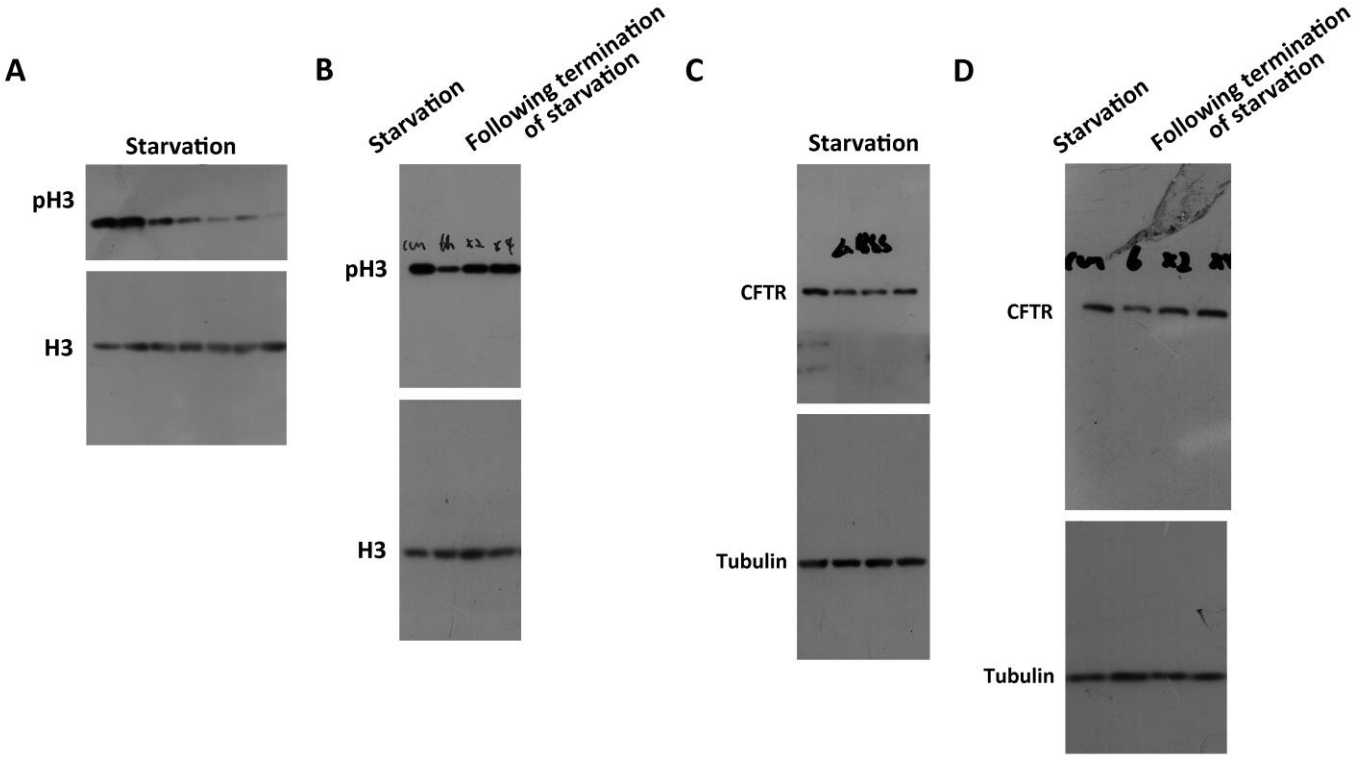


**Figure S3.** Scans of Western blots showing bands representinng pH3, H3, CFTR and tubulin, referred to **(A)** figure 1C, **(B)** figure 1E, **(C)** figure 2B and **(D)** figure 2C.

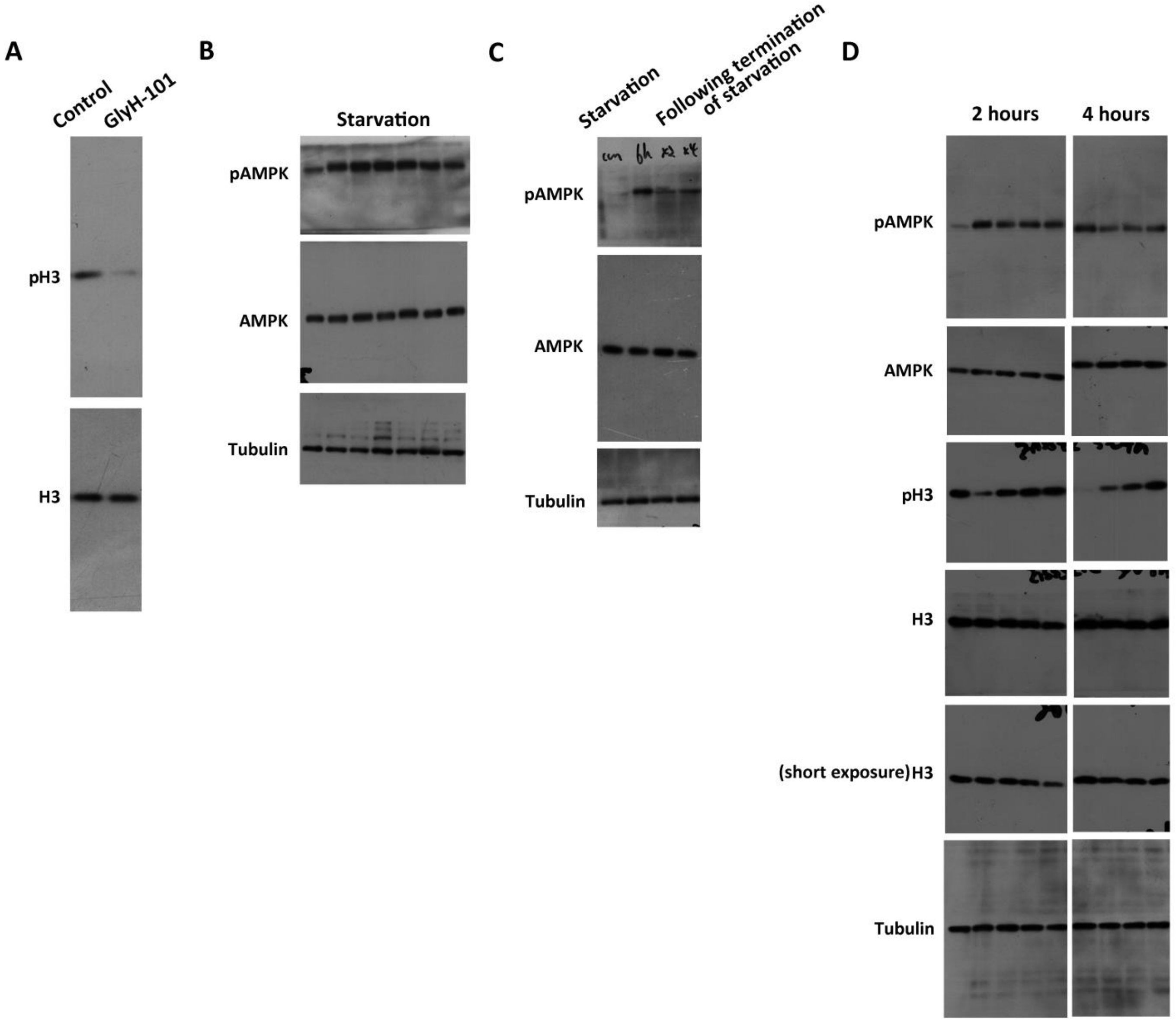


**Figure S4.** Scans of Western blots showing bands representinng pH3, H3, pAMPK, AMPK and tubulin, referred to **(A)** figure 3C, **(B)** figure 4A, **(C)** figure 4B and **(D)** figure 4C.

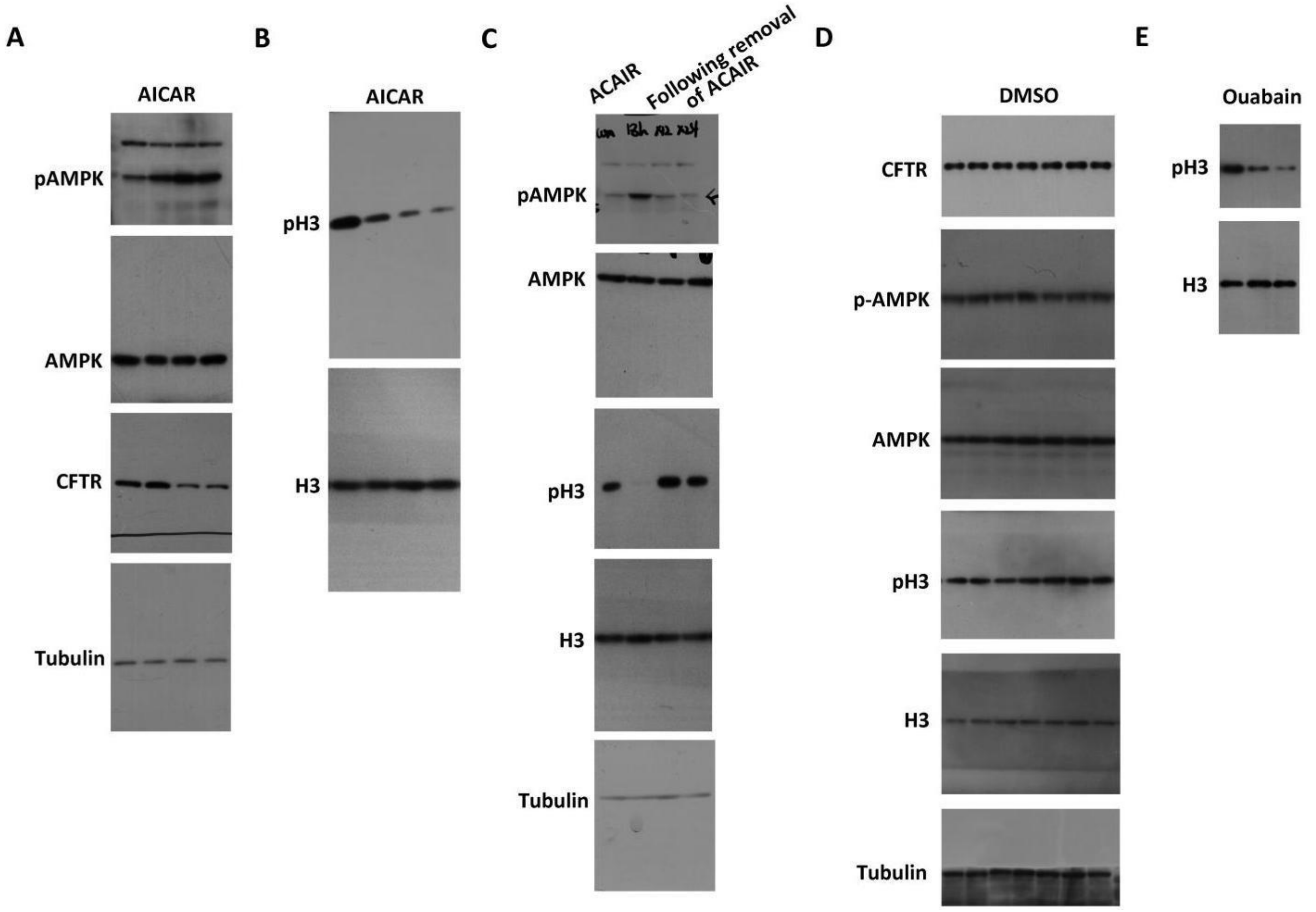


**Figure S5.** Scans of Western blots showing bands representinng pAMPK, AMPK, CFTR, pH3, H3 and tubulin, referred to **(A)** figure 5A, **(B)** figure 6B, **(C)** figure 7A, **(D)** figure S8C, **(E)** figure S10C.

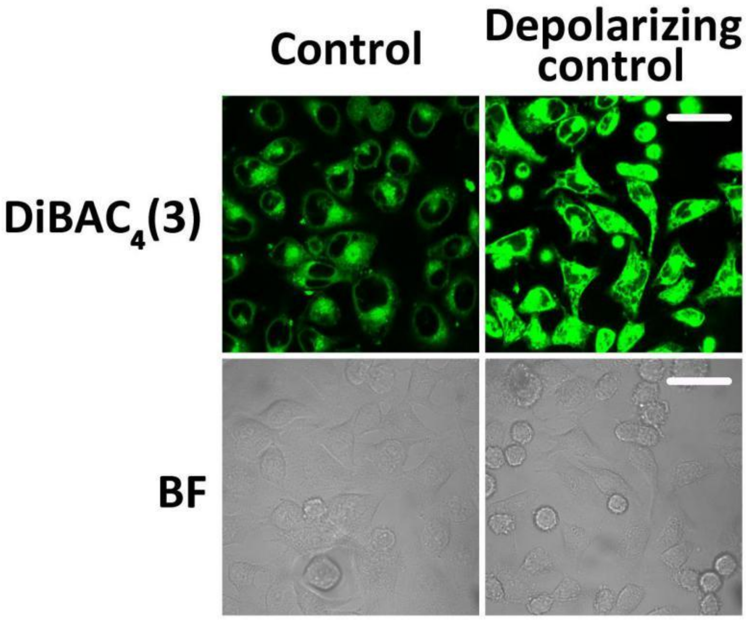


**Figure S6.** Membrane potential of MKN45 cells after depolarize treatment. Potassium gluconate (150 mM) and salinomycin (20 mM) were used for depolarization. Membrane potential was determined using a fluorescent bioelectricity reporter, DiBAC$_4$(3) (green). BF: bright field. Scale bars, 50 µm.

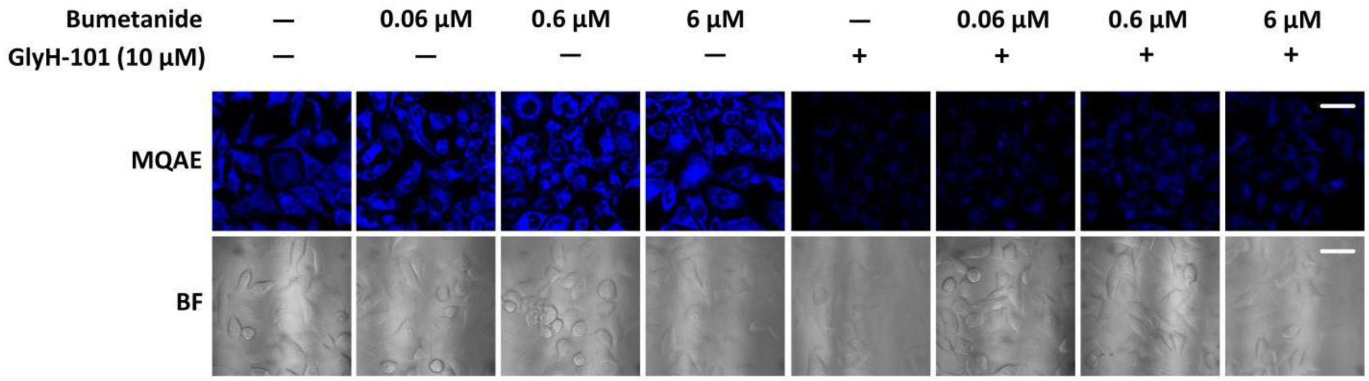


**Figure S7.** Inhibition of chloride entry using bumetanide effects intracellular chloride accumulation caused by Glyh-101. Measurement of intracellular chloride concentration in GlyH-101 (10 nM)-treated and Bumetanide (0.06 μM, 0.6 μM, 6 μM)-treated cells using the chloride-sensitive dye MQAE (blue). BF: bright field. Scale bars, 50 μm.

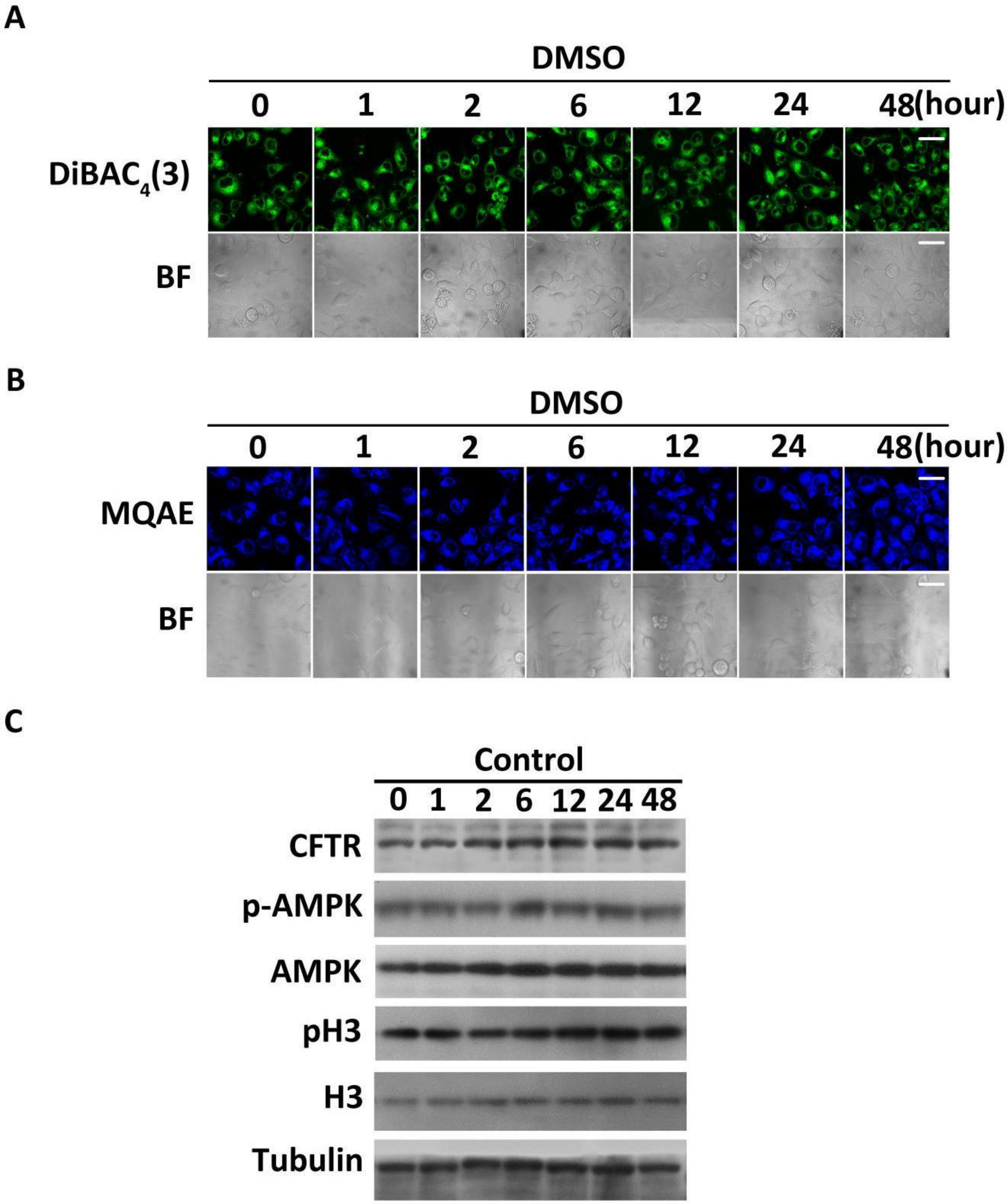


**Figure S8.** Time-dependent control in MKN45. **(A)** Membrane potential of cells treated with DMSO, detected using DiBAC$_4$(3) (green). BF: bright field. Scale bars, 50 μm. **(B)** Measurement of intracellular chloride concentration in DMSO-treated cells using the chloride-sensitive dye MQAE (blue). BF: bright field. Scale bars, 50 μm. **(C)** Western blotting analysis of phosphorylation of AMPK, H3 and expression of CFTR in DMSO-treated cells, α-Tubulin served as a loading control.

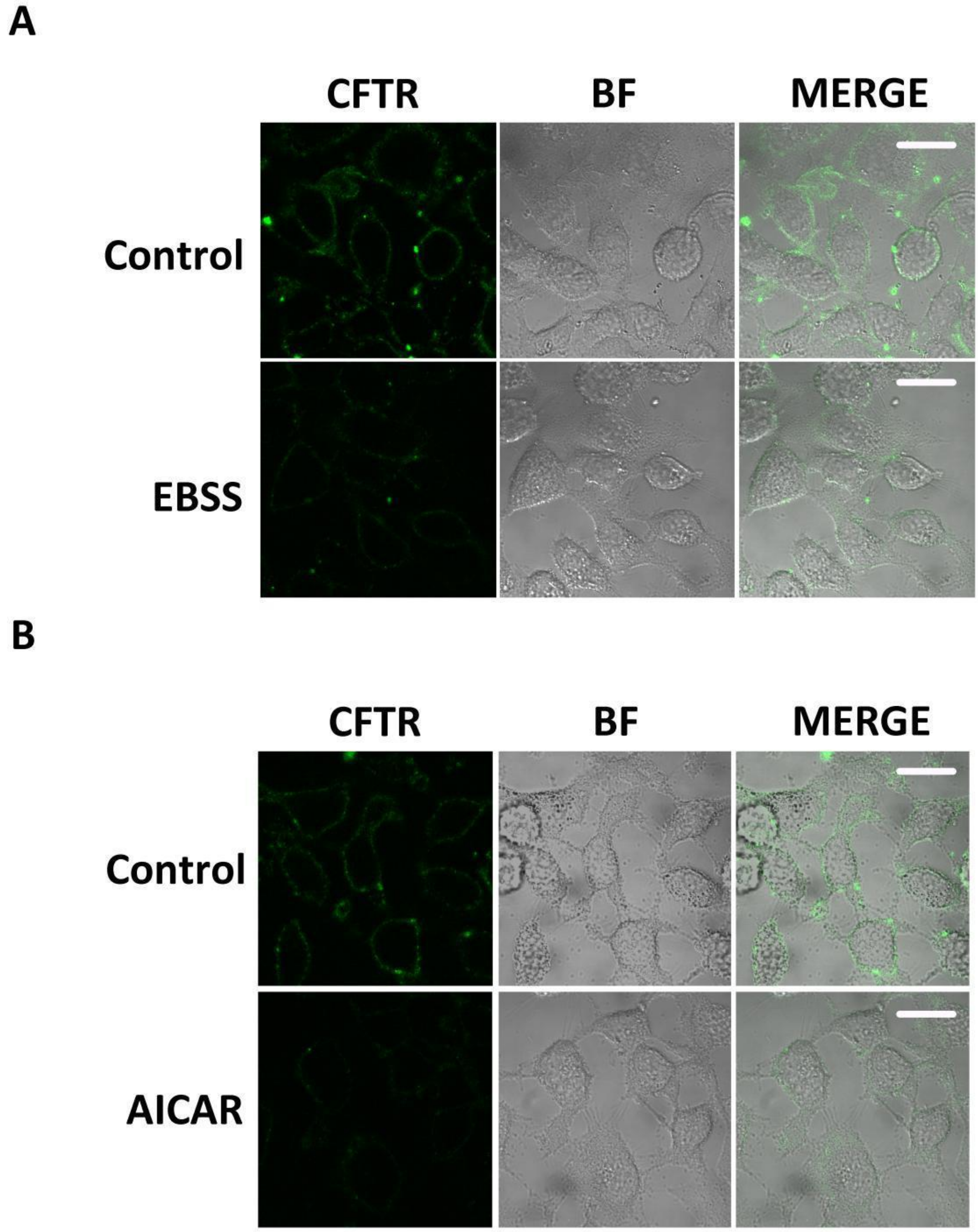


**Figure S9.** Starvation and AICAR treatment suppresses the CFTR from the plasma membrane in MKN45. Surface immunofluorescence staining analysis of CFTR expression on live cells treated with nutrient-starved for 4 hours **(A)** and AICAR for 18 hours **(B)**. Green signals reflect expression of CFTR. Scale bars, 50 µm.

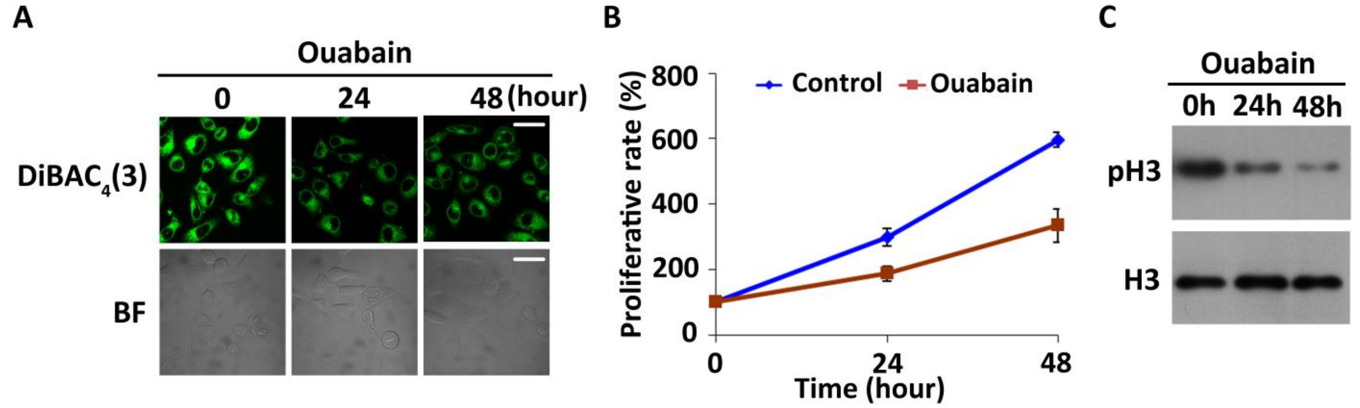


**Figure S10.** Hyperpolarization induced by Ouabain suppresses cell division in MKN45. **(A)** Membrane potential of cells treated with Ouabain (10 nM), detected using DiBAC$_4$(3) (green). BF: bright field. Scale bars, 50 µm. **(B)** Proliferation rates of Ouabain-treated (10 nM) cells were assessed by CCK-8 assay. All data are expressed as means ± SD. **(C)** Western blotting analysis of phosphorylation of H3 in Ouabain-treated (10 nM) cells, H3 served as a loading control.
